# Abnormal Tryptophan Metabolism in HIV and *Mycobacterium tuberculosis* Infection

**DOI:** 10.3389/fmicb.2021.666227

**Published:** 2021-06-28

**Authors:** Xiaolei Wang, Smriti Mehra, Deepak Kaushal, Ronald S. Veazey, Huanbin Xu

**Affiliations:** ^1^Division of Comparative Pathology, Tulane National Primate Research Center, Tulane University School of Medicine, Covington, LA, United States; ^2^Southwest National Primate Research Center, Texas Biomedical Research Institute, San Antonio, TX, United States

**Keywords:** tryptophan metabolism, indoleamine 2, 3-dioxygenase, HIV, *Mycobacterium tuberculosis*, tryptophan metabolites

## Abstract

Host metabolism has recently gained more attention for its roles in physiological functions and pathologic conditions. Of these, metabolic tryptophan disorders generate a pattern of abnormal metabolites that are implicated in various diseases. Here, we briefly highlight the recent advances regarding abnormal tryptophan metabolism in HIV and *Mycobacterium tuberculosis* infection and discuss its potential impact on immune regulation, disease progression, and neurological disorders. Finally, we also discuss the potential for metabolic tryptophan interventions toward these infectious diseases.

## Introduction

Human immunodeficiency virus (HIV) is characterized by the massive loss of CD4 + T cells, functional impairment of immune cells, disruption of the lymphoid tissues, and chronic activation (Veazey et al., 1998; Brenchley et al., 2006; Sankaran et al., 2008; Xu et al., 2013; Estes et al., 2018; Wang and Xu, 2018). Antiretroviral therapy (ART) has dramatically reduced HIV-1 replication and viremia, yet residual low-level replication-competent proviral reservoirs remain functional in a latent state, resulting in lifelong infection and viral rebound once ART is discontinued (Barouch and Deeks, 2014; Ventura, 2020). Therefore, the intact proviral reservoirs are the major obstacle in a cure for HIV infection. On the other hand, tuberculosis (TB) is caused by *Mycobacterium tuberculosis* (*Mtb*), which induces persistent pulmonary inflammation and multi-organ necrosis with a high risk of mortality and morbidity worldwide (Adigun and Singh, 2020; Kim et al., 2020). Strikingly, indoleamine 2,3-dioxygenase 1 (IDO1) activity is significantly elevated in both HIV and *Mtb* infection, correlating with AIDS and TB diseases (Favre et al., 2010; Drewes et al., 2015; Gostner et al., 2015; Jenabian et al., 2015; Routy et al., 2015; Dagenais-Lussier et al., 2016; du-Gyamfi et al., 2017; Gautam et al., 2018; van Laarhoven et al., 2018). There are three rate-limiting enzymes that catalyze tryptophan (Trp) to generate metabolites along the kynurenine (Kyn) pathway (KP), including Trp IDOs (TDO; regulator of the systemic levels of Trp), IDO1 (high enzyme activity and predominant tissue distribution), and IDO2 (proinflammatory responses, distribution in liver and neurons) (Rodriguez Cetina et al., 2017). IDO1 contributes to intestinal homeostasis (Alvarado et al., 2019), because Trp metabolites [L-Kyn and kynurenic acid (KYNA)] act as natural ligands and in signaling of aryl hydrocarbon receptor (AhR), involving tolerance in inflammation (DiNatale et al., 2010; Pallotta et al., 2011; Bessede et al., 2014). However, overexpression of IDO1 might be implicated in immunosuppression (Mandi and Vecsei, 2012; Schmidt and Schultze, 2014; Dagenais-Lussier et al., 2016) or neurotoxicity (Campbell et al., 2014; Braidy and Grant, 2017; Lovelace et al., 2017). Trp is an essential amino acid to regulate host immunity, inflammation, bacterial killing, and neurotransmission. In addition to primary KP pathway (∼95%), Trp could also be catalyzed by alternative Trp hydroxylase (TPH) that converts Trp metabolism to produce melatonin and serotonin (∼5%) as monoamine neurotransmitter for neuroprotection neurotransmitters (Claeysen et al., 2015; Bethea et al., 2017) or other metabolites as anti-inflammatory modulators (Wu et al., 2014). The KP may competitively dampen the TPH/serotonin pathway, likely resulting in preferential immunosuppressive and neurodegenerative relevance (Meltzer et al., 1998; Ruhe et al., 2007; Bethea et al., 2017). The balance of Trp metabolism is thereby critical for physiological function. Here, we summarize advances and recent findings of Trp metabolism, IDO activity, and outcomes in HIV/*Mtb* infection and discuss the possible intervention strategies in Trp metabolic abnormalities.

## Tryptophan Metabolism in the HIV/SIV Infection

**FIGURE 1:**
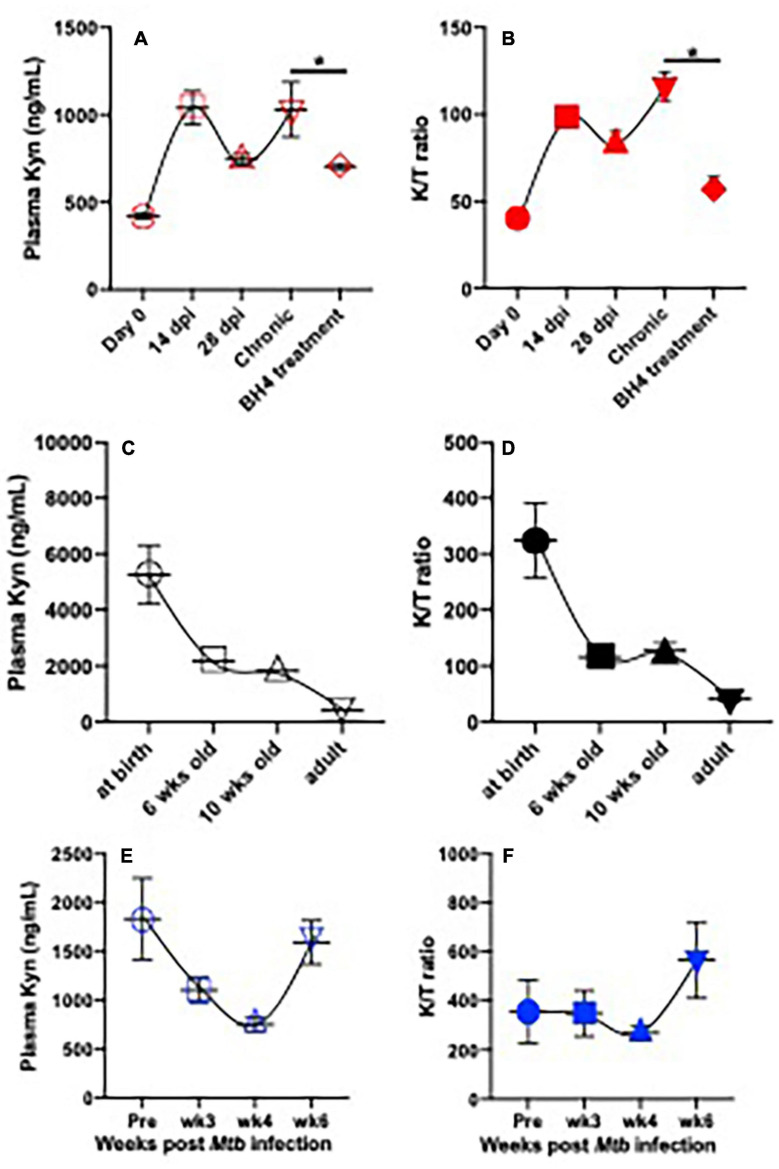
Plasma IDO activity and kynurenine metabolite in SIV-infected adult rhesus macaques on BH4 treatment and their changes with ages or in infant macaques post *Mycobacterium tuberculosis* infection. **(A,B)** Dynamics of plasma Kyn and IDO activity (K/T ratio) in adult animal post SIV infection and effects of BH4 treatment on plasma Kyn levels and IDO activity; Indian rhesus macaques (*n* = 4) were intravenously inoculated with SIVmac251 at 100TCID50. In chronic SIV infection (>3 months postinfection), macaques orally received BH4 (10 mg/kg/day, Schircks Laboratories, St. Gallen, Switzerland) for 1 month. ^∗^*p* < 0.05, compared with untreated animals at chronic stage. **(C,D)** Dynamics of plasma Kyn and K/T ratios in animals with age; **(E,F)** dynamics of plasma Kyn and K/T ratio in infant macaque post *Mtb* infection. Infant macaques (*n* = 3) were inoculated with 20 colony-forming units (CFUs) of *M. tuberculosis* CDC1551 by aerosol route. Plasma was collected to measure levels of Kyn and K/T ratio by ELISA according to instructions (ImmuSmol, Bordeaux, France). Kyn, kynurenine; Trp, tryptophan; IDO, indoleamine 2,3-dioxygenases; BH4, (6*R*)-5,6,7,8-tetrahydro-L-biopterin dihydrochloride. K/T (Kyn/Trp) ratio represents IDO activity.

HIV infection has profound effects on the immune system, as indicated by compromises in host immunity and neurological disorders (Xu et al., 2013; Eggers et al., 2017), accompanied by microbial translocation and persistent inflammation (Douek et al., 2009). In the context of HIV infection, HIV infection upregulates IDO expression by HIV tat, nef, and proinflammatory mediators (Smith et al., 2001; Schroecksnadel et al., 2007), which promote Trp degradation to KP to generate various Kyn intermediate metabolites (Huengsberg et al., 1998; Murray, 2003b). Of these, increased Kyn and its downstream metabolites may play an opposite role in infectious diseases, e.g., Kyn-associated immunosuppression (T-cell dysfunction and exhaustion, and Treg differentiation) (Curti et al., 2010; Mandi and Vecsei, 2012; Schmidt and Schultze, 2014) and dysbiosis of gut microbiota (Vujkovic-Cvijin et al., 2013) and neuroactive intermediates picolinic acid, KYNA, and nicotinamide adenine dinucleotide (NAD)-associated neuroprotective effects (Baran et al., 2000; Badawy, 2017) or 3-hydroxykynurenine/3-HK, quinolinic acid (QUIN)-mediated neurotoxicity and neurological disorders (Cervenka et al., 2017; Lovelace et al., 2017). Early initiation of ART normalizes plasma Trp catabolism and immune activation but does not improve gut mucosal dysfunction in HIV infection (Jenabian et al., 2015). Given that persistent inflammation and gut dysbiosis still occur even during long-term virologic suppression by ART (Bandera et al., 2018), elevated IDO activity and subsequent Kyn derivatives may maintain gut Th17 loss and neurocognitive dysfunction (Jenabian et al., 2013; Vujkovic-Cvijin et al., 2013; Keegan et al., 2019). In fact, ART shows mild to no impact on the changes of plasma Trp or K/T ratio (IDO activity) in people living with HIV (Byakwaga et al., 2014; Routy et al., 2015; Keegan et al., 2019). Conversely, quite a number of HIV-infected patients on ART still suffer from HIV-associated neurocognitive disorders, correlating with elevated IDO activity and Kyn production and loss of serotonin (Ferrell and Giunta, 2014; Drewes et al., 2015). Further, elevated quinolinic acid and serotonin losses in the cerebrospinal fluid (CSF) of simian immunodeficiency virus (SIV)-infected rhesus macaques are only partially resolved with ART (Drewes et al., 2015). Our preliminary data indicate that SIV infection induces plasma IDO activity and downstream metabolite Kyn throughout viral infection (Figures 1A,B). It is reported that tetrahydrobiopterin (BH4), an essential cofactor for TPH, and exogenous BH4 treatment potentially rescue T-cell responses by suppression of Kyn production (Cronin et al., 2018). Our studies also showed that BH4 feeding significantly reduced levels of Kyn and IDO activity that were typically elevated during SIV infection, yet there were no effects on the plasma viral load (data now shown). These findings suggest that HIV/SIV infection may induce elevation of both IDO activity and Kyn production, likely involved in immune regulation, yet further investigations are needed to understand the abnormalities in Trp metabolism induced by HIV/SIV infection. Abnormalities of Trp metabolism, induced by the persistent inflammation, are probably linked to pathological outcomes in HIV infection (Schroecksnadel et al., 2008; Keegan et al., 2016; Lovelace et al., 2017; Babu et al., 2019). Notably, IDO activity is the checkpoint that is positively associated with size and persistence of HIV reservoirs (Alzahrani et al., 2019; Chen et al., 2019). However, current 1-methyl-D-tryptophan (D1MT; IDO inhibitor) administration does not effectively block IDO activity and reduce downstream Kyn metabolites in SIV-infected macaques (Boasso et al., 2009; Dunham et al., 2013). Taken together, elevated IDO activity in HIV infection and lifelong ART remain a risk for many health conditions, including chronic diseases, abnormalities in Trp metabolism, and neurological diseases (Sacktor, 2018).

## Tryptophan Metabolism in Tuberculosis Disease

Converging evidence shows that both innate and adaptive immune cells play an important role in controlling *Mtb* infection (North and Jung, 2004; Podinovskaia et al., 2013). Macrophages are the sentinel immune cells and the major target cells for *Mtb* infection, which are involved in recognition, phagocytosis, pathogen digestion, and induction of different activation pathways (proinflammatory M1 and anti-inflammatory M2 phenotypes). Cytolytic granule-secreting T cell responses also coordinate to reduce bacterial burdens (Mogues et al., 2001; Lazarevic and Flynn, 2002). Increasing evidence shows that Trp metabolism affects *Mtb* growth and activity (Qualls and Murray, 2016; Collins et al., 2020), as indicated by *Mtb*-infected macrophage transcriptome profiling revealing high expression of several enzymes controlling Trp catabolism (Memari et al., 2015). During *Mtb* infection, IFN-γ-activated macrophages attempt to limit pathogen growth through Trp starvation, yet *Mtb* defends against this auxotroph threat by inducing Trp biosynthesis (Zhang et al., 2013; Berney and Berney-Meyer, 2017). In TB, granulomas formed in response to *Mtb* are immunological barriers to limit bacterial dissemination and growth (Ehlers and Schaible, 2012; Gideon et al., 2015). Notably, many metabolic events occur within the TB granuloma influencing the function of immune cells, potentially determining bacterial persistence or clearance. Increased IDO expression induced by IFN-γ in *Mtb* infection can limit intracellular bacterial growth through degradation and starvation of Trp in systemic and anatomic tissues (Cowley and Elkins, 2003; Sakai et al., 2014; Khan et al., 2016). On the other hand, IDO-catalyzing Kyn metabolite is also involved in immunosuppression to avoid overwhelming tissue injury, leading to persistent bacterial infection.

In both active and latent TB disease, Trp metabolism is highly regulated to produce Kyn. Serum IDO activity is elevated in active TB patients than latently *Mtb*-infected subjects (Weiner et al., 2012), which significantly declines in patients after standard TB treatment (Almeida et al., 2009; Suzuki et al., 2012), suggesting that IDO might be a potential target in TB disease. Therefore, IDO activity (Kyn/Trp ratio) and metabolite changes could be a predictor in the onset of TB and active TB disease (Collins et al., 2020). In addition, *Mtb* can also promote *de novo* synthesis of NAD + from Trp metabolite QUIN (Fricker et al., 2018). A nicotinamide analog, isoniazid (INH), is thus developed to be an effective anti-TB drug (Murray, 2003a), suggesting that Trp metabolites could be therapeutic targets for the *Mtb* treatment. Our previous study demonstrates the expression of IDO is highly induced in the lung of adult rhesus macaques, and that D1MT (IDO inhibitor) treatment could suppress IDO activity and reduce the bacterial burden in experimental *Mtb* infection (Gautam et al., 2018). Distinct from adult immune systems, developing infants are more vulnerable to TB infection and more prone to develop active diseases (Blusse van Oud-Alblas et al., 2002; Newton et al., 2008; Esposito et al., 2013; Roya-Pabon and Perez-Velez, 2016; Kay et al., 2018). We examined levels of plasma Kyn and IDO activity (Kyn/Trp ratio) in uninfected infants after birth. The data showed that both were higher in plasma of newborns and then rapidly declined with age, reaching a low-level set point in adults (Figures 1C,D), likely causing active immunosuppression in early life (Medzhitov et al., 2012; Kollmann et al., 2017). Strikingly, *Mtb* infection in infant animals 3 months of age did not increase Kyn production and IDO activity within 4 weeks after *Mtb* inoculation but significantly promoted IDO-catalyzing Kyn accumulation by ∼6 weeks, in concert with both elevated IDO activity and Kyn metabolites in *Mtb*-infected infant animals at this time point (Figures 1E,F), indicating that treatment with IDO inhibitors may be a viable treatment strategy to test in *Mtb*-infected infants. Although conventional anti-TB treatment may also reverse the IDO-mediated KP (Collins et al., 2020), the mechanisms at the intersection of Trp metabolism and TB *in vivo* are still unknown.

## Tryptophan Metabolism in *Mtb*/HIV Coinfection

HIV/*Mtb* coinfection places a huge burden on public health, especially in resource-limited countries. This coinfection synergistically acts to impair immunological functions, devastating multiple aspects of host immunosurveillance (Getahun et al., 2010; Du Bruyn and Wilkinson, 2016; Day et al., 2017), as indicated by macrophage as reservoir for both pathogens and impaired differentiation and function of specific T cells (Geldmacher et al., 2010; Chetty et al., 2015; Kalokhe et al., 2015; Suarez et al., 2015; Day et al., 2017), leading to death if untreated. HIV-infected individuals without ART show a more than 20-fold higher risk to develop active TB disease than HIV-uninfected patients (Lawn and Zumla, 2011), as HIV infection predisposes the host to be susceptibility to *Mtb* infection and the incidence of TB disease (Mukadi et al., 1997; Palme et al., 2002). Although ART in HIV/*Mtb* coinfected patients reduces opportunistic infections and enhances *Mtb*-specific T cell responses, it may not ameliorate TB diseases due to the paradoxical immune reconstitution inflammatory syndrome (IRIS) (Manosuthi et al., 2006; Lawn et al., 2007; Meintjes et al., 2008; Elliott et al., 2009; Namale et al., 2015). It is reported that HIV/*Mtb* coinfected patients display higher plasma IDO activity and more rapid TB disease progression from latent to active TB, than do those with TB infection alone (Collins et al., 2020), suggesting that high IDO activity and Kyn-related metabolites are still maintained at high levels in HIV/*Mtb* coinfected individuals (du-Gyamfi et al., 2017), presumably generating similar function of Kyn metabolites to the HIV or *Mtb* infection. It remains unclear that such elevated IDO activity is caused by HIV and *Mtb* infection or implicated in progressive HIV and TB disease. TB treatment could reduce IDO activity in HIV + patients with TB, indicating plasma IDO activity is a biomarker of active TB in HIV-positive patients (du-Gyamfi et al., 2017). Since D1MT treatment shows discrepant effects of IDO inhibition in *Mtb* or SIV-infected macaques (Boasso et al., 2009; Dunham et al., 2013; Gautam et al., 2018), more IDO inhibitors are expected to test for their IDO inhibition in the HIV/*Mtb* coinfection. Due to the minimal impact of ART on the KP pathway in HIV/SIV infection, it remains unknown whether treatment combined anti-HIV drugs with specific IDO inhibitor could be beneficial for containment of both pathogens in HIV/*Mtb* coinfection. Trp metabolism in HIV/*Mtb* coinfection is currently less understood.

## Interventions of Tryptophan Metabolism in HIV/*Mtb* Infection and Future Perspectives

Given the critical role of Trp metabolism in HIV/*Mtb* infection, therapeutic interventions that target this pathway may reverse the presence of, or levels of, aberrant metabolites in these infectious diseases. To prevent KP and its downstream metabolites in the abnormal Trp metabolism, IDO inhibitors, which structurally mimic Trp substrate, such as Trp analogs D1MT (indoximod, IC50 > 2.5 mM), L-1-methyl-Trp (L1MT; IC50 = 120 μM), epacadostat (IC50 = 73 nM), and navoximod (IC50 = 28 nM), linrodostat (IC50 = 3.4 nM), are applied in current clinical trials (Gunther et al., 2019). The IDO inhibition by 1-MTs *in vivo* might be ineffective and inadequate owing to their low affinity to the IDO enzyme and high dose required, as indicated by their high doses in a patient, which do not increase their serum levels (Soliman et al., 2014; Wirthgen et al., 2016; Gunther et al., 2019). Epacadostat is a higher potent IDO1 inhibitor, and oral administration of epacadostat with 100 mg twice daily reaches a plasma concentration of 0.8 μM on day 1 and 0.9 μM on day 8 (Mitchell et al., 2018). Navoximod possesses dual inhibition of IDO1 and TDO as a potent drug for absorption and bioactivity by oral administration (Ebata et al., 2020). Linrodostat treatment as IDO inhibitor could reduce Kyn concentration by up to 90% (Zhang et al., 2021). Preclinical and clinical trials with IDO inhibitors are still being investigated.

**FIGURE 2:**
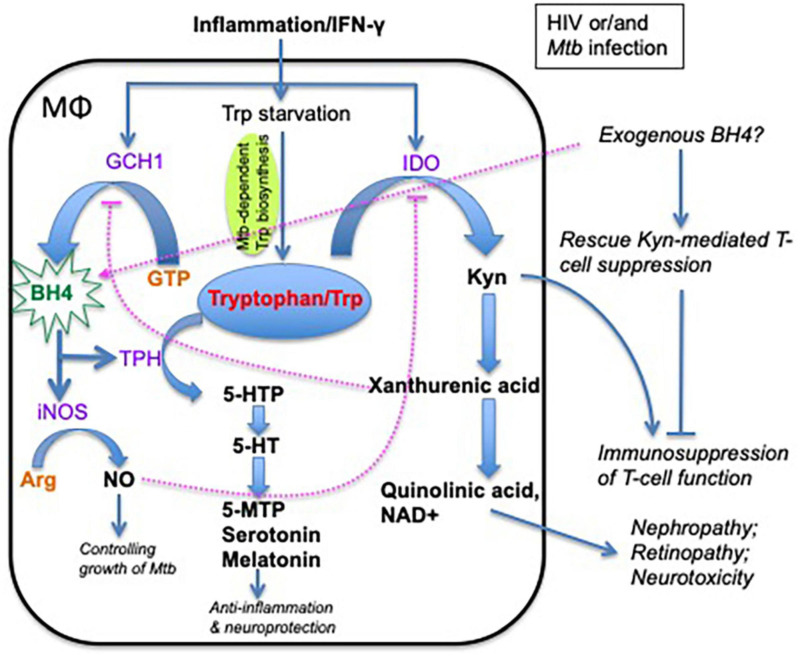
Schematic of alternate tryptophan pathways and metabolites and their effects on macrophages, inflammation, and neurofunction. Tryptophan has two major metabolic pathways: (1) indoleamine 2,3-dioxygenase (IDO) pathway, which generates kynurenine-associated immunosuppression, and (2) tryptophan hydroxylase (TPH) pathway in which the tryptophan metabolites are involved in neural function modulation and anti-inflammatory effects. Note that tryptophan metabolism plays an important role in host immunity and neurological disorders in HIV/*Mtb* infection. BH4 is an essential enzyme cofactor for NOS in NO production, and THP in the conversion of tryptophan to 5-HTP. Importantly, NO strongly inhibits IDO1 activity, and exogenous BH4 may rescue kynurenine-mediated T-cell suppression. GCH1, GTP cyclohydrolase 1; BH4, tetrahydrobiopterin (one of tryptophan hydroxylase); 5-HTP, 5-hydroxytryptophan; 5-MTP, 5-methoxytryptophan; 5-HT, 5-hydroxytryptamine (serotonin); melatonin, *N*-acetyl-5-hydroxytryptamine; TPH, tryptophan hydroxylase; IDO, indoleamine 2,3-dioxygenase; Kyn, kynurenine; NO, nitric oxide; iNOS, inducible nitric oxide synthase; NAD, nicotinamide adenine dinucleotide; D1MT, 1-methyl-D-tryptophan.

In the context of HIV/*Mtb* infection, persistent proinflammatory responses may also activate GTP cyclohydrolase 1 (GCH1) to produce BH4 (Kapatos, 2013), probably mediating bactericidal activity and IDO inhibition via nitric oxide (NO) (Thomas et al., 2007; Jamaati et al., 2017; McNeill et al., 2018), promoting serotonin production for neuroprotection (Claeysen et al., 2015; Bethea et al., 2017) or other metabolites for anti-inflammatory mechanisms (Yang et al., 2015). It would be important to know whether altering the balance of Trp metabolism between two pathways (IDO or TPH/iNOS activity) could provide an environment of effective immunity, bacterial killing, control of inflammation, and neuroprotection in HIV or/and *Mtb* infection. It is reported that treatment with exogenous BH4 can rescue T-cell function from Kyn-mediated T-cell suppression, based on the fact that *de novo* BH4 synthesis is inhibited by Kyn metabolites *per se* (Zhang et al., 2013; Haruki et al., 2016), considering that BH4 has the potential to (1) promote NO production for bacterial killing; (2) block IDO/KP-mediated immunosuppression; (3) convert Trp metabolism to the TPH/serotonin pathway for neuroprotection and anti-inflammatory effects; and finally (4) rescue viral and bacterial T-cell immune responses (Figure 2) (Cronin et al., 2018; Fanet et al., 2020; Staats Pires et al., 2020). We hypothesize that exogenous BH4 immunotherapy may be a useful strategy to modulate Trp metabolism in HIV/*Mtb*-infected patients. However, further studies in animal models are clearly required to carefully evaluate whether such agents can modulate Trp metabolism to provide a safe and effective treatment for HIV/*Mtb* coinfection.

## Author Contributions

XW wrote the manuscript. DK, SM, DK, RV, and HX revised the manuscript. All authors contributed to the article and approved the submitted version.

## Conflict of Interest

The authors declare that the research was conducted in the absence of any commercial or financial relationships that could be construed as a potential conflict of interest.

## Abbreviations

AIDS: acquired immunodeficiency syndrome
ART: antiretroviral therapy
HIV: human immunodeficiency virus
IDO: indoleamine 2,3-dioxygenase
Kyn: kynurenine
*Mtb*: *Mycobacterium tuberculosis*
SIV: simian immunodeficiency virus
TB: tuberculosis
Trp: tryptophan.
